# Energy harvesting from plants using hybrid microbial fuel cells; potential applications and future exploitation

**DOI:** 10.3389/fbioe.2024.1276176

**Published:** 2024-01-31

**Authors:** John Greenman, Robin Thorn, Neil Willey, Ioannis Ieropoulos

**Affiliations:** ^1^ School of Applied Sciences, College of Health, Science and Society, University of the West of England, Bristol, United Kingdom; ^2^ Civil, Maritime and Environmental Engineering Department, University of Southampton, Southampton, United Kingdom

**Keywords:** microbial fuel cell, MFC-plant hybrid, PhotoMFC, bioenergy harvesting, constructed artificial wetlands

## Abstract

Microbial Fuel Cells (MFC) can be fuelled using biomass derived from dead plant material and can operate on plant produced chemicals such as sugars, carbohydrates, polysaccharides and cellulose, as well as being “fed” on a regular diet of primary biomass from plants or algae. An even closer relationship can exist if algae (e.g., prokaryotic microalgae or eukaryotic and unicellular algae) can colonise the open to air cathode chambers of MFCs driving photosynthesis, producing a high redox gradient due to the oxygenic phase of collective algal cells. The hybrid system is symbiotic; the conditions within the cathodic chamber favour the growth of microalgae whilst the increased redox and production of oxygen by the algae, favour a more powerful cathode giving a higher maximum voltage and power to the photo-microbial fuel cell, which can ultimately be harvested for a range of end-user applications. MFCs can utilise a wide range of plant derived materials including detritus, plant composts, rhizodeposits, root exudates, dead or dying macro- or microalgae, via Soil-based Microbial Fuel Cells, Sediment Microbial Fuel Cells, Plant-based microbial fuel cells, floating artificial islands and constructed artificial wetlands. This review provides a perspective on this aspect of the technology as yet another attribute of the benevolent Bioelectrochemical Systems.

## Introduction

**FIGURE 1:**
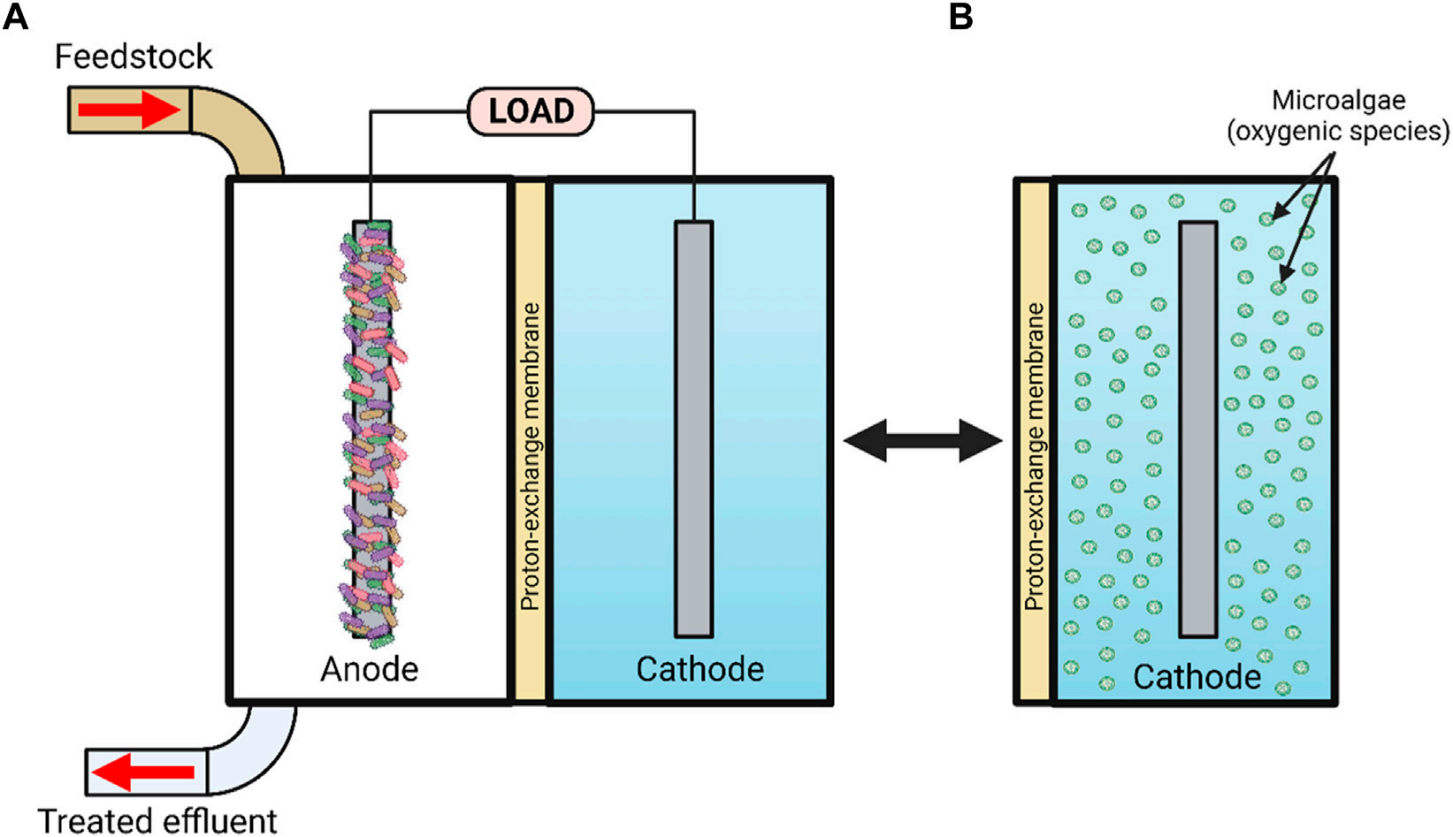
Schematic representation of **(A)** a microbial fuel cell [MFC] and **(B)** a micro-algal photomicrobial fuel cell [pMFC] with oxygenic species present within the cathode. Created with BioRender.com.

Microbial fuel cell (MFC) technology is an emerging green technology, capable of generating clean electrical energy through exploitation of environmentally sustainable microbial processes (Gajda et al., 2018). It has taken over 100 years to develop the discovery of MFC by Potter (1911) into a viable technology for the 21st century. A typical MFC consists of an anodic and a cathodic compartment separated by a proton exchange membrane (PEM) (see Figure 1), whereby the organic material entering the anodic compartment (feedstock) is digested by various types of microbial species around the anode (Chae et al., 2009). Some types of heterotrophic fermenting bacteria are capable of degrading a wide range of substrates including macromolecular substrates, e.g., starch, chitin, pectin, cellulose, hemicellulose and lignocellulose, and ferment them into carboxylic acids, e.g., formic, acetic, lactic, propionic and butyric, whilst electroactive species utilize carboxylic acids and degrade them into CO_2_, reducing power (NADH) and electrons via anaerobic respiration, using the anode as an end terminal electron acceptor. Cations such as protons migrate to the cathodic chamber via the cation exchange membrane (CEM), where they react with electrons and oxidising agents such as ferricyanide, nitrate, persulphate, permanganate, triiodide, hydrogen peroxide or oxygen from the atmosphere to produce electricity (Logan et al., 2006; Ucar et al., 2017).

MFC are inexpensive; research and development has moved towards the use of oxygen for oxidation rather than high-cost maintenance chemicals (e.g., ferricyanide or potassium permanganate), thereby avoiding costly cathodic catalysts, e.g., platinum, which can be replaced by biocathodes or inexpensive transition metal catalysts or nano carbon (graphene) components. In addition, the membrane/separator is replaced with low-cost ceramics. Use of ceramics in MFCs was first reported by Park and Zeikus (2003) incorporating graphite electrodes and a proton permeable porcelain separator, a similar case of which was described by Seo et al. (2009). Moreover, an off-the-shelf 400 mL sized ceramic pot MFC demonstrated a power output of 16.8 W/m^3^ and showed that a low-cost, abundant material might be able to change the course of research and accelerate the advancement of MFC research (Behera et al., 2010a; Behera et al., 2010b). Several other groups of workers now use ceramic materials and find this material to have unique thermal, chemical and mechanical characteristics offering a great advantage over polymeric membranes such as Nafion^®^. For a review on the use of ceramics in microbial fuel cells the reader is referred to Winfield et al. (2016) and Yousefi et al. (2017).

MFCs possess wide substrate specificity and have mainly been used to treat waste streams composed of multipart mixes of distinctive substrate classes, for example, brewery waste (Angosto et al., 2015), urine (Ieropoulos et al., 2012), sewage sludge (Jiang et al., 2009), and wastes that are contaminated by heavy metals, for example, landfill leachate (Greenman et al., 2009). MFCs can also utilize volatile compounds including methane (for a review see Kondaveeti et al., 2019), toluene (Zhang et al., 2018) and mixed volatiles such as benzene, toluene, ethylbenzene, and xylene (BTEX) (Zhang et al., 2019). In fact, MFCs seem to perform well when provided with virtually anything organic, including petroleum hydrocarbons (Morris and Jin, 2008; Zhang et al., 2017) and polycyclic aromatic hydrocarbons (PAHs) such as, naphthalene, phenanthrene, chrysene, fluorene, pyrene, anthracene, acenaphthene and acenaphthylene (Gambino et al., 2017). In addition, algal biomass has even been effectively utilised to enhance electricity generation (Rashid et al., 2013).

**FIGURE 2:**
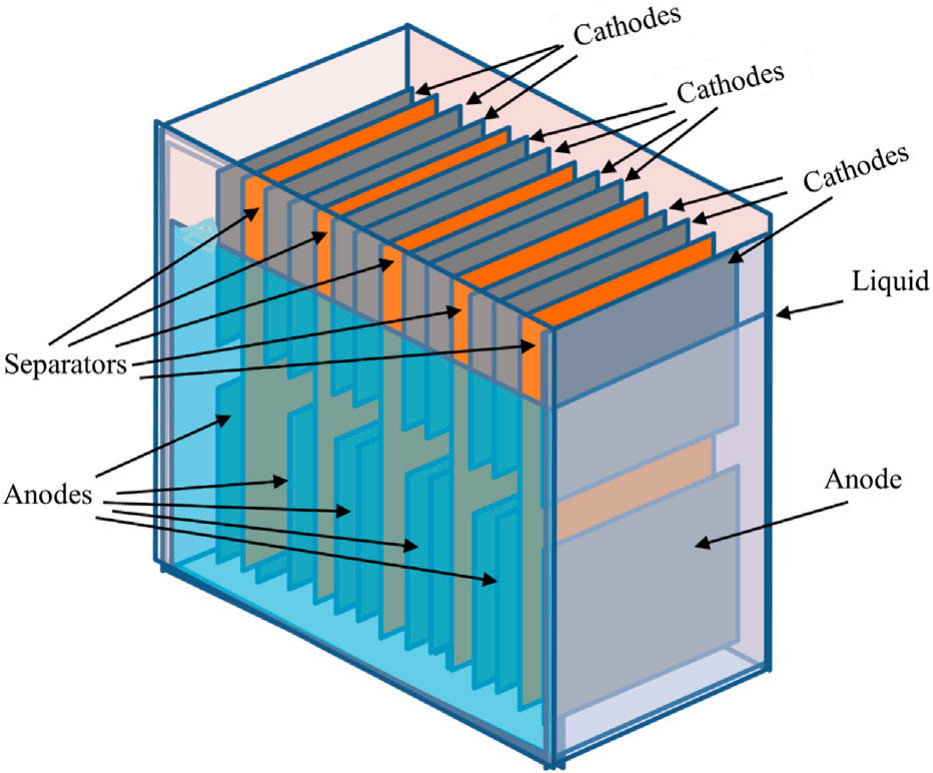
Sediment-based, or “self-stratifying” MFC. Note that the liquid level does not fully cover the cathodes, the tops of which are exposed to air. Fluid input and output pipes are not shown for reasons of clarity.

**FIGURE 3:**
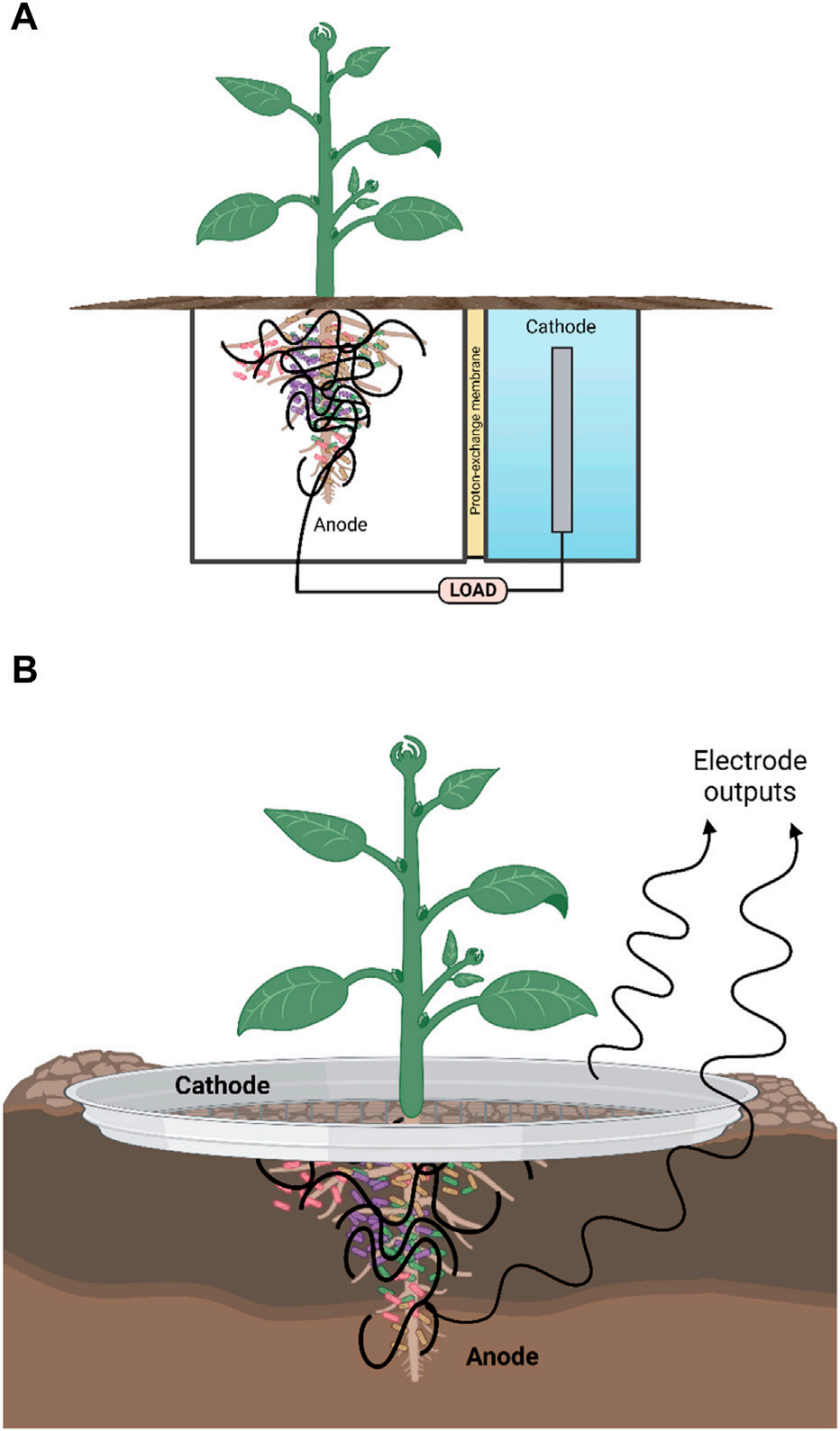
Schematic showing two types of plant microbial fuel cell (PMFC). **(A)** Double chamber separated by proton exchange membrane (PEM). The PEM can be fabricated from ceramic membranes. **(B)** Membrane-less. For both types, the voltage reached by the PMFC depends on the redox gradient that forms between the (anaerobic) anode around the roots and soil and the cathode which consists of a round ring or sheet of conductive material (metal, e.g., galvanised steel or carbon mesh) “placed” at the interface of the soil-air (aerobic) electrode. The cells around the anode use anaerobic respiration to produce electrons and protons, whereby the power required to maintain the voltage is the microbial reducing power. Created with BioRender.com.

Of particular interest and the central focus of this review is the ability of MFCs to utilize plant material and plant products such as cellulose (Ahmad et al., 2013). Recently, different MFC variants have been developed and optimised according to the proposed main fuel source and ultimate purpose of the fuel cell (e.g., power output and bioremediation capabilities). These variant examples include: membrane-less MFCs (Figure 2) mud MFC, soil MFC, sediment MFC, floating island and constructed wetland MFC, photosynthetic (algal) MFC, plant-MFC and biophotovoltaic cells (BPV-MFC) (Lea-Smith et al., 2016; Fischer, 2018; Boas et al., 2022). The latter system uses photoautotrophic cyanobacteria in the anodic chamber of the MFC, which must be illuminated by an appropriate light source. It is challenging to relate diverse reports, as standardization of the growth method, biomass production, and the systems set-up is non-uniform. Due to the speciation and materials used for BPV-MFCs, experimental power output levels described to date are orders of magnitude lower than conventional microbial fuel cells. However, if compared appropriately with conventional photovoltaics, taking into account the low amount of light input required, then BPV-MFCs are ideal for indoor use (Saar et al., 2018). A plant microbial fuel cell (PMFC) (Figure 3) is a promising modification of the MFC that exploits unique plant-microbe relationships, as seen within the rhizosphere region of a plant, converting solar energy into bioelectricity indirectly by utilizing the rhizo-deposits as an aqueous fuel. The *in situ* bioelectricity from biomass production by the plants via rhizo-deposits, instead of the supply of outside substrates, make this technology distinctive from conventional MFCs.

## Sediment-based microbial fuel cells

Sediment MFCs (SMFCs), also referred to as benthic MFCs in some cases (Li and Yu, 2015) are deployed in a natural system or where there is less engineering management (e.g., a constructed wetland). Unlike reactor MFCs that have a clear boundary between the anode and the cathode by using membranes or separators, SMFCs rely on a naturally occurring oxygen gradient to separate the anode and the cathode. Not all MFC architectures use membrane separators; some are truly membrane-less and this includes a group of MFC called sediment-based MFCs. In these cases, the anode is placed at the bottom of the reaction chamber covered in sediment, and the cathode is placed at the air interface, partly in the bulk fluid and partly open to air (see Figure 2; Dumas et al., 2007; Walter et al., 2016). Instead of a separating material between the anode and cathode electrodes, a polarity difference naturally occurs due to redox gradients that form between the aerobic oxygen enriched surface (high redox), and the low redox of the anaerobic sediment, as demonstrated by the classic Winogradsky column (Corner, 1992; Esteban et al., 2015) and proved in the MFC context by Park and Zeikus (2003). Potential applications for sediment type MFC include powering *in situ* maritime/environmental and weather telemetry instruments (Tender et al., 2008; Arias-Thode et al., 2017). More recently, sediment based MFC, or sometimes called “self-stratifying” MFC have been used in field trials for the treatment of wastewater including urine (Walter et al., 2018; Walter et al., 2019; Walter et al., 2020).

**FIGURE 4:**
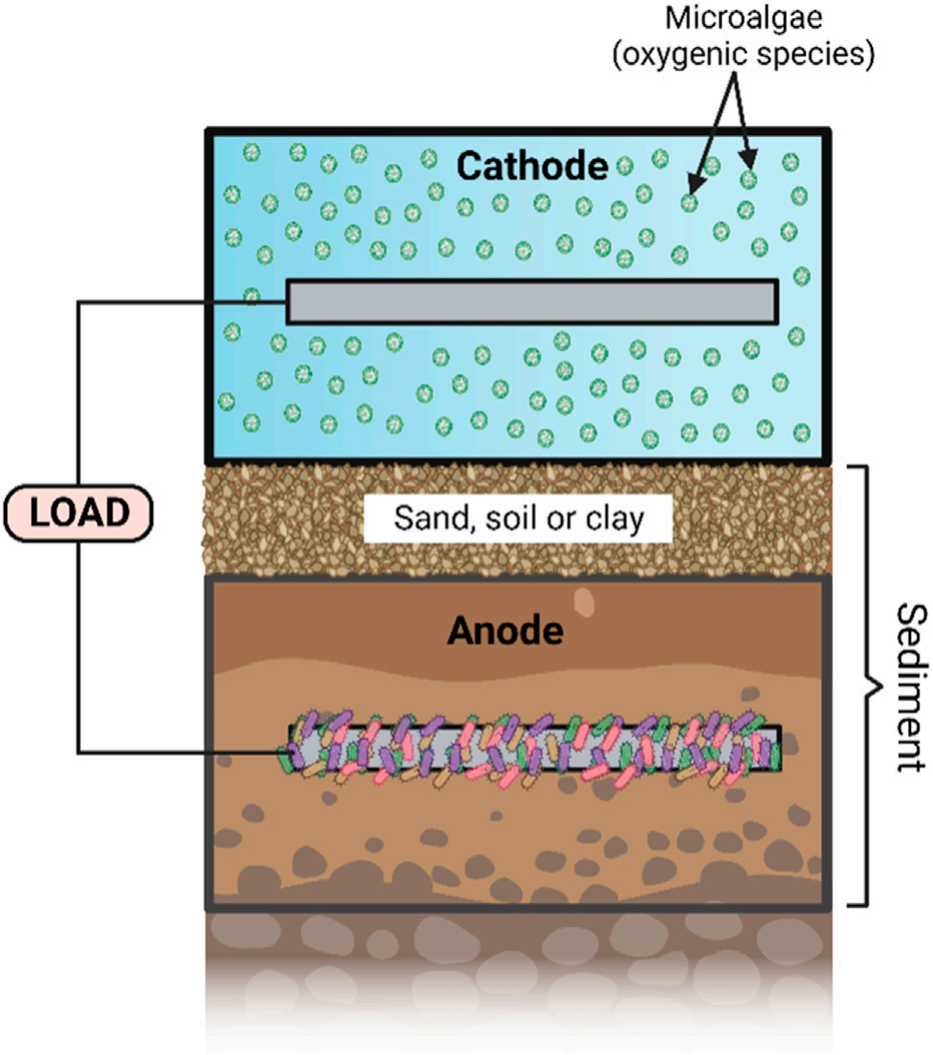
Diagram illustrating a photo-sediment microbial fuel cell (PSMFC). Created with BioRender.com.

S-MFC is a potential substitute for renewable energy sources for specific applications, for example, it can generate electricity to power sensors, devices, lighting and storage while requiring little maintenance. Implementing photosynthetic organisms like algae around the cathode (Figure 4) has been shown to be an effective method to improve power output (Xiao and He, 2014) and should be focussed on for upscaled applications.

## Soil-based microbial fuel cells

Organic rich soil contains high quantities of diverse microbes, including electrogenic bacteria (Wang et al., 2019) and species that can reduce, transform or oxidise nitrogen compounds as well as heterotrophic species that utilise organic carbon. In a soil-based MFC, soil acts as the nutrient-rich anodic medium in addition to the microbial inoculum as well as the proton selective exchange membrane (PEM). With regard to nutrients, then soils are full of complex nutrients that have accumulated from plant and animal material decay. The soil-based Microbial Fuel Cells (SMFC) use natural bacteria or secreted enzymes to break down the fuel, typically to generate electricity from the soil. In MFCs, bacteria and enzymes act as biocatalysts to produce electricity (Lan et al., 2010; Siddiqui and Pathrikar, 2013). The solid-state materials like soil compost are preferred to liquid matrix to overcome the problem of fuel loss via volatilisation (e.g., of ammonia, or hydrogen sulphide) or fuel dilution by flooding or fluidic loss by leakage, causing unstable behaviour.

When bioanodes go into wet soil, providing there is a suitable air- or oxygenated water-cathode, then the system will work as an MFC. Most semi-submerged MFCs utilise rhizodeposits, but some patches of soil (fertilised by urine or animal manure) may have an even higher nutrient load, similar to that existing in an anaerobic digester or cesspit bioreactor. The cathode rests on top of the soil where it is exposed to air and oxygen, whilst the anode is positioned into the ground, deep within the soil (Gupta et al., 2023).

## Plant-based microbial fuel cells

Plant MFC (PMFC) were originally designed with the idea of interfacing a plant with the anodic compartment of an MFC, as source of substrate for microbial metabolism (Strik et al., 2008). In a Plant-MFC, the plants continue to develop and simultaneously yield sufficient organic matter for microbes to grow and generate bioelectricity. Thus, this technology represents a green energy platform with concomitant biomass and electricity generation.

One advantage of plant based MFCs is that they operate in the absence of a PEM separator, because the soil supporting the plants growth also enables ion transport, taking the role of the PEM membrane within classical MFCs, reducing the complexity of the MFC design and material costs. However, the lack of a membrane within PMFCs does reduce performance, possibly due to the diffusion of oxygen from the atmosphere or from the roots to the anode, hindering the growth, start-up time and bioactivity of the anaerobic microflora, resulting in a lower efficiency of the utilisation of the organic matter excreted by the plants (Helder et al., 2013a). The amount of oxygen accessible to root cells is important for healthy plant growth and crop harvest. Root cells without oxygen are restricted by the quantity of sugar they can burn and the amount of nutrients and water that they can absorb (Roblero et al., 2020). In soils without oxygen, some plants create an aerobic micro-environment around their roots. The movement of oxygen from the roots into the soil is described as radial oxygen loss (ROL) (Jiménez et al., 2021). The extent to which plants can oxygenate their rhizosphere depends on the species. ROL might therefore also inhibit the rate by which rhizodeposits are utilised by the anode of a PMFC. Further studies also demonstrated that electrodes inserted directly into the earth below the plants became coated with soil particles giving less surface area for biofilm to colonise thus leading to a decrease in PMFC output (Helder et al., 2013b). There are mechanisms for improving PMFC performance, for example, adding a conductive carbon material such as activated carbon or carbon pellets to the soil increases the soil conductivity to improve the power output with minimum impact on plant viability (Sudirjo et al., 2018).

Plant photosynthetic pathways can be classified to C3, C4, and CAM (Sayed et al., 2021). C4 plants are the most favoured because they convert CO_2_ to a 4-carbon sugar compound that is transported to the stroma (the inner space of chloroplasts) where it is decarboxylated, providing carbon dioxide for the reactions of the Calvin-Bensen cycle (Schreier and Hibberd, 2019). In addition, C4 plants produce a considerable amount of bioenergy, and are well adapted to hot and dry conditions. C4 plants also produce higher amounts of rhizodeposition as a substrate for the microorganisms near the root, enhancing the power regeneration in plant-based microbial fuel cells (PMFC) (Lambers et al., 2009; Sayed et al., 2021). C4 plants would be expected to have a higher rate of rhizo-deposits and consequently a higher power output when integrated into a PMFC. Moreover, given that C4 plants are highly efficacious in a dry, hot atmosphere, this adaption would enable use within a wider range of environments (Sales et al., 2021). An alternative is to use CAM plants (Nobel, 1991) which are also adapted to dry and arid regions, but tend to be slower growing, possibly due to their property of fixing CO_2_ at night time as well as in light conditions (Hartsock and Nobel, 1976).

Wetland plants including reed manna grass (*Glyceria maxima*; Strik et al., 2008), rice plants, (*Oryza sativa*; Ueoka et al., 2016), common cordgrass (*Spartina anglica*; Timmers et al., 2010) and giant weed (*Arundo donax*; Helder et al., 2010) are the most commonly used plant species for PMFC. Selecting the most suitable plant is an obvious way to improve electricity output. However, many other species have been investigated for different purposes, these being primarily production of bioelectricity and wastewater treatment within PMFCs: *Pennisetum setaceum* (Chiranjeevi et al., 2012), *Cyperus involucratus* (Klaisongkram and Holasut, 2015), *Lolium perenne* (Habibul et al., 2016), *Eichhornia crassipes* (Mohan et al., 2010), *Acorus calamus* (Yan et al., 2015), *Ipomoea aquatica* (Liu et al., 2013), *Typha latifolia* (Oon et al., 2015), *Echinochloa glabrescens* (Bombelli et al., 2013) and *Canna indica* (Lu et al., 2015). Ultimately, the efficiency of these systems is driven by the production and release of exudates or rhizodeposits by these plants, which is determined by sunlight (Shin et al., 2008), temperature (Junttila, 1980) and growth stage (Wu et al., 2013). In addition, bryophytes have been used in pMFC. The moss species *Physcomitrella patens* was used in MFC by Bombelli et al. (2016) and this was reported to generate sufficient electrical power to energise a commercial radio receiver or an environmental sensor.

## Composition of root exudates and rhizodeposits—impacts on PMFC performance

Rhizodeposits consist of exudates, mucilage and sloughed-off tissues and root cells, many of which lyse their contents in the vicinity (Dennis et al., 2010). Rhizodeposits tend to be insoluble, whereas root exudates tend to be water soluble. The relationship between root morphology, photosynthetic efficiency and amount of root exudates is an important feature of plant MFC which determines the power output. Root exudate has long been known to contain a wide range of, generally soluble, organic compounds (Dennis et al., 2010), with recent studies beginning to establish their functional diversity (e.g., Ma et al., 2022). Important categories of biochemicals exuded include amino acids (all types have been found), sugars and oligosaccharides (over a dozen types of sugar), a wide range of organic acids, many longer chain fatty acids (linoleic, linolenic, oleic, palmitic, stearic), sterols (campesterol, cholesterol, sitosterol, stigmasterol), growth factors and vitamins, a range of proteins (including enzymes such as amylase, invertase, peroxidase, phenolase, acid phosphatase, alkaline phosphatase, polygalacturonase, protease), flavonones, nucleotides (adenine, guanine, uridine and cytidine) and many other biologically active compounds (e.g., auxins, scopoletin, HCN, glucosides, reducing compounds, ethanol, glycinebetaine, inositol, and myo-inositol-like compounds, dihydro-quinone, sorgoleone, isothiocyanates). In addition, gaseous molecules (e.g., CO_2_, H_2_, H^+^, OH^−^, HCO_3_^−^, CH_4_) can also be emitted from roots. For photosynthesis products, around 30%–50% within pasture plants, and 20%–30% within cereals, ends up within the root system (Kuzyakov and Domanski, 2000). For cereals, about half of this carbon remains in the roots, whilst about a third is liberated from the rhizosphere by cell respiration (roots or microbes) within a few days, and the remaining fraction is incorporated into microbial biomass and soil organic matter (Kuzyakov and Domanski, 2000). The estimates of carbon economies within plants give only approximate figures because the reported values vary considerably, and the matter remains controversial. What is not in doubt is that the performance of a PMFC will be improved with better rhizodeposition and higher rates of rhizoexudates through condition optimisation coupled with choice of suitable plants (Strik et al., 2008).

## Photo-microbial fuel cells—microalgae

Photo-microbial fuel cell systems utilise fast growing microalgae in the cathode whilst removing organic matter through the anode. *Chlorella vulgaris* in the cathodic chamber has been studied by a number of workers (Powell et al., 2009; Wang et al., 2010; Wu et al., 2013). All these authors noted that *Chlorella* cells could serve as electron acceptors improving the MFC power output by improving the cathode. An integrated photobioelectrochemical cell whereby an MFC core unit is immersed within a photobioreactor has been described by Xiao et al. (2012). This showed that algal photosynthesis under white light illumination produced higher levels of dissolved O_2_ for the cathode than was produced by using mechanical aeration in the dark. More importantly, the microalgae showed their ability to remove N and P nutrients in wastewater in addition to supplying oxygen to the cathodes (Campos et al., 2014; He et al., 2014). One system was reported to achieve organic removal (>90%), ammonium removal (nearly 98%) and phosphate removal (82%) yet produce sufficient energy to theoretically meet the requirement of energy consumption by this system (Kakarla and Min, 2014). Following mixed culture inoculum of a PMFC, one study showed cyanobacteria *Leptolyngbya* and the green alga *Acutodesmus* as becoming the dominant photoautotrophs in the cathode suspension (Tse et al., 2016). A mixed community of fast-growing oxygenic algae was also used by Walter et al. (2013). These workers showed that a filamentous cyanobacteria (*Anabaena cylindrica*) could facilitate the anchoring of two other strains in the cellulose matrix, *Chlorella pyrenoidosa* and the cyanobacterium *Synechococcus leopoliensis*.

Algal biomass production in conjunction with wastewater treatment and power generation within a fully biotic Photo Microbial Fuel Cell (pMFC) has been demonstrated by Gajda et al. (2015) who designed and built a system whereby the anaerobic biofilm in the anodic half-cell generated current and new (bacterial) biomass, whereas the phototrophic biofilm on the cathode provided oxygen for the Oxygen Reduction Reaction (ORR) and generated algal biomass. The benefit of photo-microbial fuel cells (pMFC) is that following removal of organic matter, the liquid is ready to supply N, P, K and other inorganic essential elements as the nutrient source for the microalgae in the cathodes, which can fix CO_2_ and utilise and remove the N, P, K through growth and replication. The new microbial biomass can then be used either as a product (e.g., animal food, biodiesel or biochar), or be directly fed back into the anode side, along with the original waste stream to act as a substrate for the anodic microbial processes (and consequent power generation). Growth of algal biomass in the cathode increased the charge transfer and power output which in turn activated the cation crossover from the anodic chamber to the cathode as a closed loop recycling machine. This system represents the simplest biotechnology for complete re-cycling of all the main elements (C, H, O, N, P, S, Mg, K and trace elements) important in supporting life (Greenman et al., 2019).

## Biophotovoltaic devices

Growing photosynthetic microalgae or cyanobacteria attached as biofilms in the anodic compartment of MFC have also been attempted (for a review see Fischer, 2018; Tschörtner et al., 2019). Such systems are described as biophotovoltaic cells (Wey et al., 2019). A microfluidic biophotovoltaic device that did not require membranes or mediators has been described (Bombelli et al., 2015). This used Synechocystis sp. PCC 6803 cells which were injected and allowed to settle on the anode to conduct electrons to the anode directly without the need for a mediator. Power densities reaching above 100 mW m^−2^ were recorded for chlorophyll concentration of 100 μM using white light. However, in general, electron production at the anode by photosynthetic algae is not particularly successful, possibly due to oxygen production by the algae.

## Scale-up and novel approaches towards PMFC real-world application

The application of conventional PMFCs coupled with rhizodeposits can be limited by environmental factors and the size and type of plant species utilised. It is now generally accepted that a large volume MFC (e.g., a 500 mL chamber volume) is less power dense than a small scale equivalent total volume MFC (e.g., 10 × 50 mL, or 100 × 5 mL) (Ieropoulos et al., 2008). The small scale MFC can be mass manufactured, being made entirely from carbon-based electrodes and ceramic body or printed chassis. The modular approach to MFC stacks can mean that collectives of MFC can be rapidly constructed, enabling the end-user to make sufficient MFC stacks for useful electrical work and/or bioremediation.

## Plant-stem-MFCs and plant fuel cells

A relatively new concept has been trialled with regard to plant-MFCs; a device that can directly generate continuous bioelectricity from the plant stem rather than the roots (Lu et al., 2020). The novel PMFC coupled with the plant stems produced more stable and continuous bioelectricity (without diurnal oscillatory behaviour) as well as a much shorter start-up period compared with a conventional control PMFC. The stem-coupled PMFCs produced bioelectricity for over 40 days. Moreover, *Populus alba* coupled PMFC showed higher power output (7.61 mW m^−2^) compared to *Pachira macrocarpa* (3.60 mW m^−2^). Additionally, the researchers studied the response of the novel PMFC to different substrate concentrations observing that the cell voltage increased following addition (injection) of moderate substrate concentrations. Moreover, a commensal relationship was formed between the plant and the anodic microorganisms. The plant stem-associated PMFC can extend the working conditions without being confined by the growth environment of aquatic plants, and broaden the range of plant species that can be coupled with PMFCs.

In addition to stem-coupled PMFC other methods of extracting green electricity have been described which do not impede plant growth. These could be described simply as plant fuel cells (PFC) because they work *without* the intervention of microorganisms. For example, a method has been published (Concepcion et al., 2023) described as an *in vivo* stem bioelectricity production system which has been shown to be capable of extracting electrical power from dragon fruit cactus trees (*Hylocereus undatus*). The method relies on the placement and penetration of anode and cathode fine pin electrodes directly pinned into the plant tissues. The authors determined the optimum distance of electrode placement of two silver-coated copper pin-type anodes and cathodes for maximum bioelectricity extraction through intercellular penetration of the pins across (a) vascular bundles and (b) inter-parenchymal cells. The authors incorporated a cradle-to-gate Life Cycle Assessment methodology to properly account for the environmental impacts of the two intercellular penetration approaches. Highest electrical energy abstraction (58.9 Joules) throughout the 30-day experiment occurred using inter-parenchymal cell penetration which surpassed the yield across the vascular bundles, which only yielded 13.9 Joules. An electrode distance of 4.488 inches produced the highest yield of harnessed bioelectricity while incurring no significant damage and causing fewer environmental impacts. Experiments included bioelectricity measurements using unoptimized electrode placement, and then optimising the distances of the two pin electrodes, one inserted across the plant stem and the other submerged under the soil and near the plant roots. This green electricity is sustainable in a way that it is continuously extracted without directly affecting the biological growth of its source, which in this case was the dragon fruit plant. Based on 1–5-inch electrode distance sweeping with the copper electrode fixed in soil, both the intercellular penetrations across the vascular bundle (icVB) and inter-parenchymal cellular region (iPC) exhibited strong positive polynomial correlations (*R* ^2^ = 0.8233 for icVB, *R* ^2^ = 0.9302 for iPC) between electrical potential and electrode distance.

## Hydroponics and their integration with MFCs

The idea that plants can grow without soil by getting their nutrients directly from water is an old idea obtained from the early observation of plants that grow in oceans, lakes and rivers. Egyptian hieroglyphics dating back to several hundred years BC show plants being grown in water along the Nile without soil. However, the Hanging Gardens of Babylon were the first known example of plants grown without soil, built approximately 2,600 years ago in Babylonia (Mesopotamia, now modern-day Iraq) and the plants were believed to have been watered via a chain pull system carrying water up from the Euphrates River and allowing it to trickle slowly down each step of the garden (Folds, 2018). The Aztecs of Central America in the 10th and 11th centuries developed an ingenious method of utilizing the concepts of hydroponics by building rafts of rushes and reeds, joining the rafts together creating floating islands of plants upon Lake Tenochtitlan in Mexico. The floating gardens were called *chinampas.* The plants would grow and direct their roots through the reed bundles of the raft and into the nutrient-rich water of the lake (Debangshi, 2021). Marco Polo whilst visiting China in the late 13th century witnessed similar “floating gardens” where rice was grown, (Rinaldi, 2016). The earliest work to be published on growing terrestrial plants without soil was the 1627 book *Sylva Sylvarum* or “A Natural History” by Sir Francis Bacon (British scientist, philosopher and politician). The work was printed a year after his death. As a result of his work, water culture in Britain became a popular research technique (Singer, 2021). For example, in 1699, John Woodward (an early scientist and fellow of the Royal Society of England) was one of the first humans to mix together water and soil to use as a root medium. He was therefore probably the first person to make hydroponic plant food and understand that plants absorb soluble nutrients from soil and water. By 1842 a list of nine recently discovered elements, believed to be essential to plant growth had been identified. In the 1850s Jean Baptiste Boussingault, a French scientist performed experiments with inert unreactive growing media and established the elemental needs for plant growth in terms of H, C, O, N and other mineral elements. He concluded that although water was essential for plant growth so were a number of mineral elements which he identified along with their proportions required for optimum plant growth. In the 1860s the German scientist Julius von Sachs, (professor of Botany at the University of Würzburg) published the first standard formulation for a mixed solution of nutrients that could be solubilised in water and would make plants grow effectively (Folds, 2018). This represented the foundation of nutri-culture. This early work showed that normal plant growth could be realised by submersing plant roots in an aqueous solution of mixed compounds comprised from the element’s N, P, S, K, Ca and Mg.

The word “hydroponics” was created in 1924 by Dr. William F Gericke of the University of California. It was used to describe crops growing in media without soil by using nutrient–enriched water indoors and outdoors. Before 1924, hydroponics was referred to as aquaculture, chemiculture or nutriculture. In 1938, Berkeley scientists Dennis Hoagland and Daniel Arnon published “The Water Culture Method for Growing Plants without Soil” which is now generally thought to be one of the most significant texts ever published about hydroponics. Many of the mineral nutrient solutions they developed are still being used today (e.g., Hoagland solution) (Folds, 2018). The commercial use of hydroponics has increased enormously over the last 50 years; during the same period gardeners and small-scale growers have accelerated the use of hydroponics to cultivate vegetables and flowers in their own homes and this continues to increase in popularity. More recently hydroponics has become progressively important due to climate change (global warming), desertification, and water shortages which are becoming increasingly significant.

**FIGURE 5:**
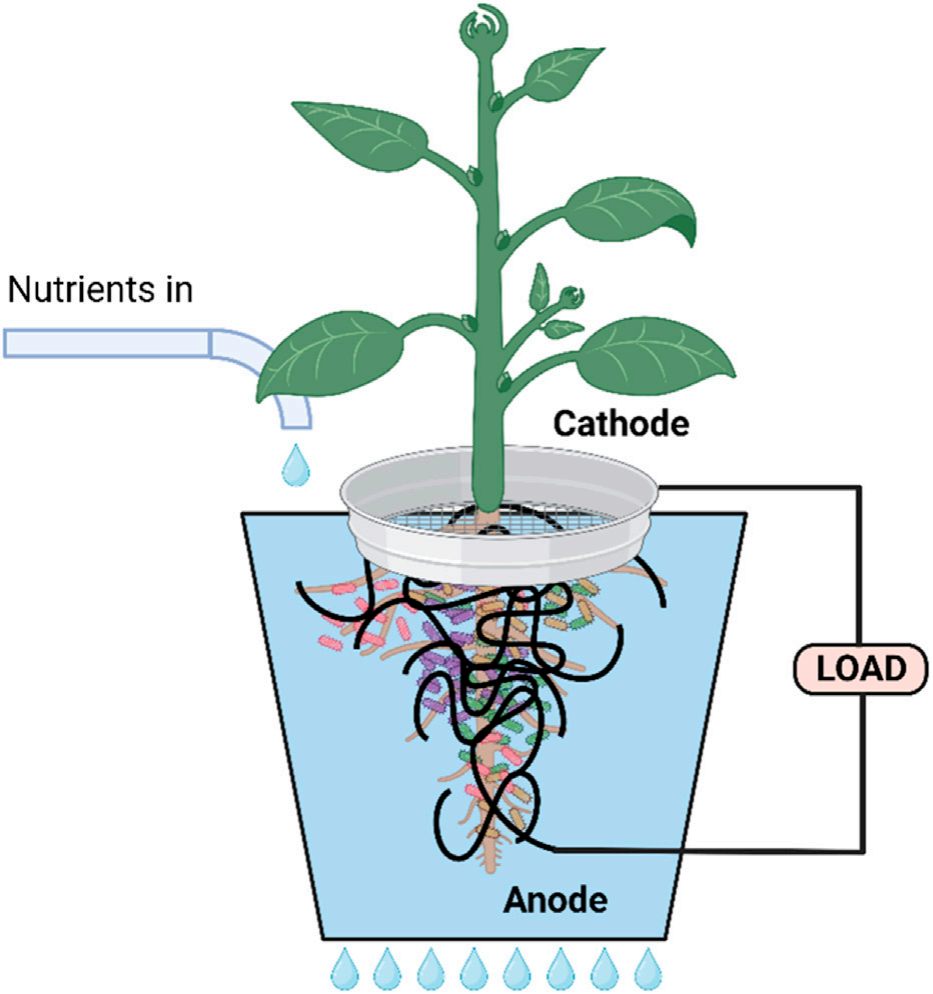
Diagram illustrating a single unit drip feeding hydroponic system. Created with BioRender.com.

A number of workers (Khuman et al., 2020; Yadav et al., 2020; Paucar and Sato, 2022) have described the integration of microbial fuel cells with hydroponics (Figure 5). Yadav et al. (2020) described the use of a novel integrated drip hydroponics-microbial fuel cell system. This consisted of influent and effluent ducts along with ten reactor units. Each unit hosted a lemongrass sapling planted in a cocopeat bed matrix, a graphite cathode around the stem (exposed to air) and an anode (around the root). COD reduction was measured after 3 h operation in batch recirculation mode, and shown to reach 72% ± 2.4% COD, 83% ± 1.1% phosphate, and 35% ± 4.2% ammonia removal efficiencies. These efficiencies increased significantly after 12 h operation. The system also yielded low but continuous levels of power and plant biomass output. The simple but efficient system design, scaled up by addition of a greater number of units could offer an easy-to-implement approach for hydroponic and wastewater treatment at the household and small community levels.

The work by Paucar and Sato (2022) was to develop a new integrated MFC-hydroponic system to generate electricity whilst concurrently degrading organic pollutants (i.e., COD) in hydroponic wastewater and simultaneously removing nitrogen (N) and phosphorus (P) as well as produce edible plants. The MFC-hydroponic system produced a power density of 250.7 mW/m^2^. Both power density and phosphorous recovery increased by approximately 19% and 7.5%, respectively in the presence of *Allium tuberosum* compared to control MFC without the plant. Khuman et al. (2020) showed the advantages of using ceramic separators in MFC, of a type likely to be used in wastewater treatment and/or hydroponic systems and/or constructed wetlands.

## Artificial floating islands and MFC

**FIGURE 6:**
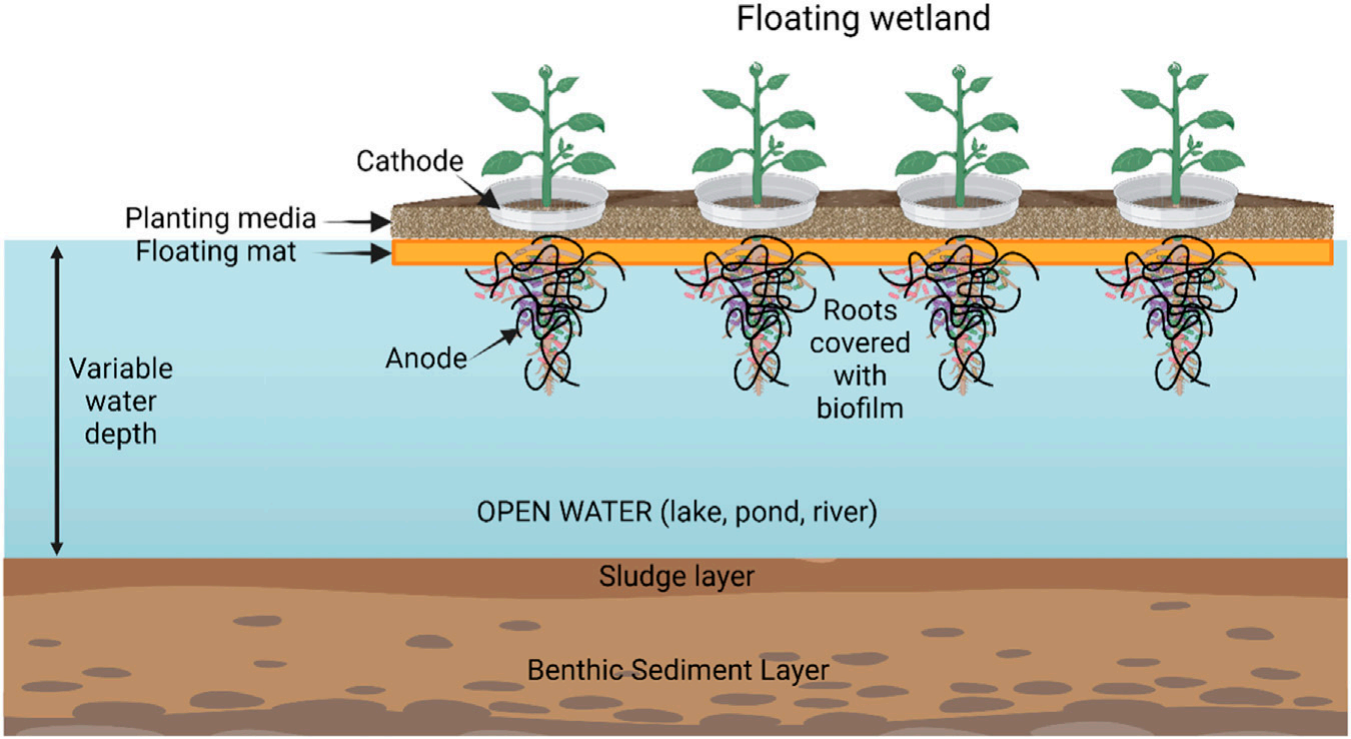
Schematic of an artificial floating island with coupled MFCs. Created with BioRender.com.

Artificial floating islands (AFIs) are a variant type of wetland treatment process for water quality purification (Figure 6). Their main applications are for the elimination of organic nutrients and COD (chemical oxygen demand) on watercourses (lakes, ponds, rivers) although they are also used for removing heavy metals (Fang and Achal, 2019). The aquatic ecology (plants, animals and microbes) in AFIs can be useful by themselves for water decontamination and purification. Addition of MFC’s improves the removal of organic matter which in turn improves the growth of the primary biomass (plants and algae).

The first publication linking MFCs to free-floating objects is probably the description of a floating type of MFC capable of treating contaminated water (An et al., 2009). In this study, the base of the anodic vessel was left open to the aquatic environment and the cathode was exposed to air above the water surface. MFC open circuit voltages reached around 0.4–0.5 V. In closed circuit mode the current reached 0.25 mA with a maximum power density of 8 mW/m^2^. Huang et al. (2012) described a floating MFC designed to float and operate in the ocean. The power output performance was evaluated over 153 days and shown to gradually increase from close to zero to a maximum value of 390 mW/m^3^ at 125 days. Compared with a sediment type of MFC, the floating MFC (installed in a buoy), was not limited by the depth of the ocean and has the potential to supply electrical power to low-power electronic devices at distant or secluded locations. Pasternak et al. (2017) constructed a floating MFC that was self-powered and capable of sensing Biological Oxygen Demand (BOD) for online water quality monitoring that could float on pools of contaminated water. A study of the long-term feasibility of in-field floating MFC, used for both monitoring anoxic wastewater and for energy harvesting was carried out by Cristiani et al. (2019) who used two types of MFC (flat and tubular). Both types of floating MFCs were shown to be able to provide electric power for years, albeit with oscillations, and despite the low concentration of organic matter in the wastewater (from denitrification tanks). The results clearly demonstrated the viability of the MFC system as a monitoring and energy harvesting system in a real environment. There was sufficient power to guarantee energy autonomy, despite the energy required for daily remote transmission of radio signals from the tested system.

A study of an ecological floating bed combined with a microbial fuel cell (MFC) was carried out by Yang et al. (2021) using four aquatic plants (windmill grass, goldfish algae, water hyacinth and water spinach). When plants were introduced into the MFC the internal resistance of the system was reduced by 21%–68% and the electrical power generation increased by 26%–63%. The coupled system enhanced the removal efficiency of NH_4_^+^-N and TN by 2.54%–16.40% and 2.91%–16.86%, respectively. The *water spinach* realised the greatest performance for both nitrogen removal and electricity generation.

The idea of a floating treatment wetland (FTW) (i.e., a floating island) combined with sediment microbial fuel cells (SMFC’s) was investigated by Shen et al. (2022). The authors constructed a novel FTW-SMFC system with vertical floating biocathodes and studied the performance and mechanisms of plant, substrate, and a bio-electrochemical system for low-strength surface water treatment. The results revealed that by combining plants with SMFCs workers could increase the power density by 32.9%–42.5%. The data also showed that the presence of both plants and substrates together significantly improved the removal of nitrogen (N) and phosphate (P), with removal efficiency increasing by 8.3%–27.8% and 3.5%–13.9% for N and P, respectively under SMFC electrochemical treatment. The authors concluded that by combining FTW with SMFC an economical, effective, and environmentally sustainable system is the combined result for energy recovery, remediation of contaminated sediments, and wastewater treatment.

## Constructed wetlands and MFCs

Constructed wetlands are built as a method to treat wastewaters, leachates, mine drainage, stormwater run-off and sludge dewatering (Vymazal, 2014a). They do this through a mixture of chemical, physical and biological processes (Vymazal, 2014b; Wu et al., 2015). Their popularity has increased over the last two decades because of their relatively low cost in terms of installation, operation and maintenance and their success and sustainability at cleaning liquid waste (Ghrabi et al., 2011). Plants have been united with MFCs on the basis of two established processes: firstly, the accumulation of organic compounds in the rhizosphere of living plants and secondly, the ability of soil microorganisms to generate electricity from organic compounds in MFCs (Strik et al., 2008). The organic feedstock in a PMFC may be totally derived from the plants rhizodeposits. However, in a constructed wetland MFC the nutrients are derived from both rhizodeposits and additional wastewater, the aim being to treat the latter. Both constructed wetlands and MFC are important for degrading organic matter and the two technologies working together can be described as being synergistic or protocooperative, each assisting the other to do the job (Wetser et al., 2015; Wetser et al., 2017). The wetland MFC used are typically (a) continuous up-flow (b) continuous upflow with aeration in cathode area; (c) continuous upflow with different spacing of the anodes and cathode; (d) simultaneous upflow–downflow; (e) horizontal flow with effluent recirculation. For a detailed review of wetland MFC the reader is referred to Doherty et al. (2015).

It is postulated that by introducing a colonised anode electrode into the rhizosphere of wetland plants, a competition for carbon and electrons can be invoked between electrogenic bacteria and methanotrophic archaea. This theory was tested and results show considerable reduction in methane production, due to the anaerobic microflora containing less methanogens and more methanotrophic species. Many different types of methanotrophic species have been isolated and formally characterized over the past 50 years (Whittenbury et al., 1970). There are many formally described genera known, including hundreds of species in the class Alphaproteobacteria (genus *Methylocella, Methylocapsa* and *Methyloferula*)*,* Gammaproteobacteria*,* family *Methylococcaceae* (genus *Methylomonas, Methylobacter, Methylococcus, Methylomicrobium, Methylosphaera, Methylocaldum, Methylosarcina, Methylothermus, Methylohalobius, Methylogaea, Methylosoma, Methylomarinum, Methylovulum, Crenothrix, Clonothrix*), and in the class of *Verrucomicrobia*, including two species: *Methylacidiphilum fumariolicum* and *Methylacidiphilum kamchatkensis*. They include Gram-positive and Gram-negative species as well as aerobic or anaerobic types and they can be isolated from a wide range of environments; marine, terrestrial and from the gut of humans and animals. Some species are able to colonise the anodes of MFC enabling the fuel cell to produce electrical power from gas streams of methane (McAnulty et al., 2017) using methane as the sole or main carbon-energy substrate. In mixed culture MFC a consortium of aerobic methanotrophs enriched by air was grown within the cathode biofilm, which produced intermediate metabolites (e.g., formate and acetate) that (in a single compartment MFC) served as substrates for *Geobacter* in the anodic biofilm (Chen and Smith, 2018).

## Conclusion

To this day, MFCs are still considered by many as insufficiently powerful, however this is based on findings from lab-based studies, often with limited materials or conditions and not fully optimised for power output; new materials, enhanced with additive nanoparticles are already demonstrating significant improvements in power output performance and this is expected to continue. When space is not limited, it is conceivable to build large-scale MFC stacks, in which case these may well provide sufficient energy to satisfy human demand, even before any further material developments. Moreover, if there is a large land space for growing plants and they are all suitable for plant-MFC, then despite their lack of efficiency and low power, they can still be collectively worthwhile around a farm or large garden. Anywhere that is available for interfacing aerobic and anaerobic environments with water and a nutrient supply will be equally useful for both plants and MFC as stacks. A stack of a few hundred PMFC should be sufficient to slow-charge batteries over days in order to supply (via the battery) high power, albeit for a shorter period of time or burst of action by energising mechatronics as robotic responders to changes in conditions; controlling the plant MFC’s and using them as a way to sense the changes in recycling conditions (aka MFC sensing). As with plants, soil-based MFC are also inefficient when it comes to producing electricity because the Soil-MFC encapsulates a relatively large volume of soil filled anodic chamber with electrodes a relatively long distance away from each other. Even if the soil MFC is relatively inefficient, it may not matter for the purpose of removing organic matter by cold oxidation (through anodic microbial processing). For example, soil based MFC could be used to help re-cycle organic material (e.g., cattle run-off) where production of electricity is less important than the rate of uptake and oxidation of organic substrate.

If the purpose is to build a machine that can produce electricity from primary biomass, then by far the most efficient (and powerful) would be to use a stack of small-scale photo-microbial fuel cell systems, utilising fast growing microalgae in the cathode whilst removing organic matter through the anode. The main outcome of these photo-microbial fuel cells is full organic and inorganic recycling [of C, N, P, Mg, Ca, CO_2_, O_2_, H_2_O], clean-up of waste-streams and generation of electrical power. In robots the electrical power could be used to control and turn on/off motors, pumps, switches, to control flow rates and flow-stream composition. In MFC/PMFC, the higher the power output, the faster the rate of recycling.

## Future prospects

Although PMFC are already used for a limited number of applications, to become more widely adopted as a green technology, more research is required to optimise their performance. In terms of plant science, the choice of suitable plants based upon morphology (overall size) and physiology, especially in terms of quality and quantity of rhizodeposition and exudation that can occur will clearly need to be an area of future research interest. For example, the potentiality of C3, C4 and CAM plants need to be investigated within an identical PMFC system in order to compare their performance in terms of their efficiency for the desired end-user requirements. Selection of species and cultivars of plants with particular exudates may be possible as well as using optimised physicochemical conditions (e.g., pH) to control the exudation rate. Breeding/GM or gene editing plants to increase exudates/type of exudate and improve the operation of the PMFC may also be possible. In terms of the microbiology, the types and strains of microbes in the rhizosphere-soil consortium are critical to PMFC performance. Therefore, there is a need to find new strains that are electrically active and better adapted to the anodic system within PMFC configurations. This could include engineered organisms that are better at utilising the available plant derived substrates (i.e., have a wide substrate specificity) or others that are better adapted to the anodic biofilm environment, have faster growth rates (consequently resulting in higher power output) or are more efficient in terms of electrical conductance from the cell to the anode. In environmental engineering, the bioremediation capabilities of MFC (e.g., for water treatment) are as important as the production of bioenergy and soil-based MFC or PMFC can play a key role in helping with heavy metals removal and degradation of toxic organics. Consequently, chemical engineering will be concerned with fabricating better electrodes to produce favourable electrochemistry while electrical engineering improvements can be made to maximise power output or chemical degradation by combinations of MFC in cascades or stacks joined in series or parallel.
